# A Rare Case of Idiopathic Noncirrhotic Portal Hypertension in a Young Patient

**DOI:** 10.7759/cureus.80322

**Published:** 2025-03-10

**Authors:** Ahmed Ali Aziz, Muhammad Ali Aziz, Muhammad Amir, Rehan Shah, Ijlal Akbar Ali

**Affiliations:** 1 Internal Medicine, INTEGRIS Health Baptist Medical Center, Oklahoma City, USA; 2 Internal Medicine, University of Kentucky College of Medicine, Lexington, USA; 3 Transplant Hepatology, INTEGRIS Baptist Medical Center, Oklahoma City, USA; 4 Internal Medicine - Rheumatology, Bayonne Medical Center, Bayonne, USA; 5 Digestive Diseases and Nutrition, University of Oklahoma Health Sciences Center, Oklahoma City, USA

**Keywords:** cirrhosis of the liver, esophagogastroduodenoscopy (egd), idiopathic noncirrhotic portal hypertension, transjugular intrahepatic portosystemic shunt (tips), variceal hemorrhage, colonoscopy

## Abstract

The most common cause of portal hypertension (PH) is liver cirrhosis. When symptoms of PH develop in noncirrhotic patients secondary to hepatic or systemic disorders, it is termed as noncirrhotic portal hypertension (NCPH) while idiopathic noncirrhotic portal hypertension (INCPH) is the term for PH present without any identifiable underlying cause. INCPH is a diagnosis of exclusion when all other causes of liver cirrhosis have been ruled out. A liver biopsy is required to diagnose INCPH. Presuming that PH is secondary to cirrhosis when no liver biopsy is performed is not always true. The incidence and prevalence of INCPH is increasing, especially in developed countries. We present a rare case of INCPH and portal vein thrombosis in a young 23-year-old male with no significant past medical history and no underlying hepatic or systemic disease.

## Introduction

Portal hypertension (PH) is defined as a portal venous pressure gradient between the portal vein and inferior vena cava of greater than 5 mmHg [1]. Liver Cirrhosis is the most common cause of PH [2, 3]. If PH develops in noncirrhotic patients secondary to underlying systemic or hepatic disorder it is termed as noncirrhotic portal hypertension (NCPH) while if PH is present without any identifiable underlying cause it is termed as idiopathic noncirrhotic portal hypertension (INCPH) [4]. Idiopathic noncirrhotic portal hypertension (INCPH) is a rare disorder [5]. Approximately 70% of patients with INCPH present with gastrointestinal bleeding secondary to esophageal varices or portal hypertensive gastropathy. Some patients might have abdominal distension and splenomegaly. Hepatic encephalopathy might be present but is usually a rare presentation [6]. Laboratory tests often reveal a preserved liver function. Imaging studies such as abdominal ultrasound or computed tomography (CT) scan reveal signs of PH without liver cirrhosis [6]. A liver biopsy is the gold standard for diagnosing INCPH to rule out any hepatic causes of PH, such as cirrhosis [6].

We present a rare case of INCPH in a 23-year-old male with no past medical history who presented with melena and hematemesis. Imaging performed was concerning for PH and portal vein thrombosis (PVT). An extensive workup to identify a systemic or hepatic cause of PH and PVT was negative. He was eventually diagnosed with INCPH and underwent esophagogastroduodenoscopy (EGD) that showed large esophageal varices that were banded. He was started on anticoagulation and follow-up EGD showed persistent large esophageal varices. He later required transjugular intrahepatic portosystemic shunt (TIPS) procedure due to esophageal and gastric varices refractory to endoscopic band ligation.

## Case presentation

A 23-year-old Caucasian male with no significant past medical history presented with diffuse abdominal pain, bloody vomiting, and black tarry stools ongoing for 2 days. He denied abdominal distension, yellowing of skin, scleral icterus, any history of alcohol consumption, non-steroidal anti-inflammatory drug (NSAID) use or any history of liver disease. On presentation his vital signs were stable. On physical exam, his abdomen was soft, non-distended, and non-tender to palpation with normal bowel sounds. His comprehensive metabolic panel (CMP), International Normalized Ratio (INR), prothrombin time (PT), partial thromboplastin time (PTT) and ammonia levels were within normal limits. His white blood cell (WBC) count was 5.0 K/uL, hemoglobin was 8.2 g/dL, hematocrit was 24%, and platelet count was 88 K/uL (Table 1). His previous hemoglobin on record approximately 6 months ago was normal (14.4 g/dL).

**Table 1:** Laboratory results on initial admission

Lab Parameters (units)	Reference Range	Result
Sodium (mmol/L)	135 – 145	140
Potassium (mmol/L)	3.5 – 5.5	3.8
Chloride (mmol/L)	96 – 106	99
Blood Urea Nitrogen (mg/dL)	6 – 24	14
Creatinine (mg/dL)	0.7 – 1.3	0.9
Bicarbonate (mmol/L)	22 – 29	23
Glucose (mg/dL)	70 - 100	98
Aspartate Aminotransferase (U/L)	13 – 39	13
Alanine Aminotransferase (U/L)	7 – 52	11
Alkaline Phosphatase (U/L)	34 – 104	51
Lactate (mmol/L)	0.5 – 2.0	1.1
White Blood Cells (k/uL)	3.8 – 10.2	5.0
Hemoglobin (g/dL)	12.9 – 16.7	8.2
Hematocrit (%)	39.2 – 48.8	24.0
Platelets (k/uL)	150 – 450	88
International Normalized Ratio (INR)	0.8 - 1.1	1.1
Partial thromboplastin time (seconds)	25 – 35	27
Prothrombin Time (seconds)	11 – 13.5	13

**Figure 1:**
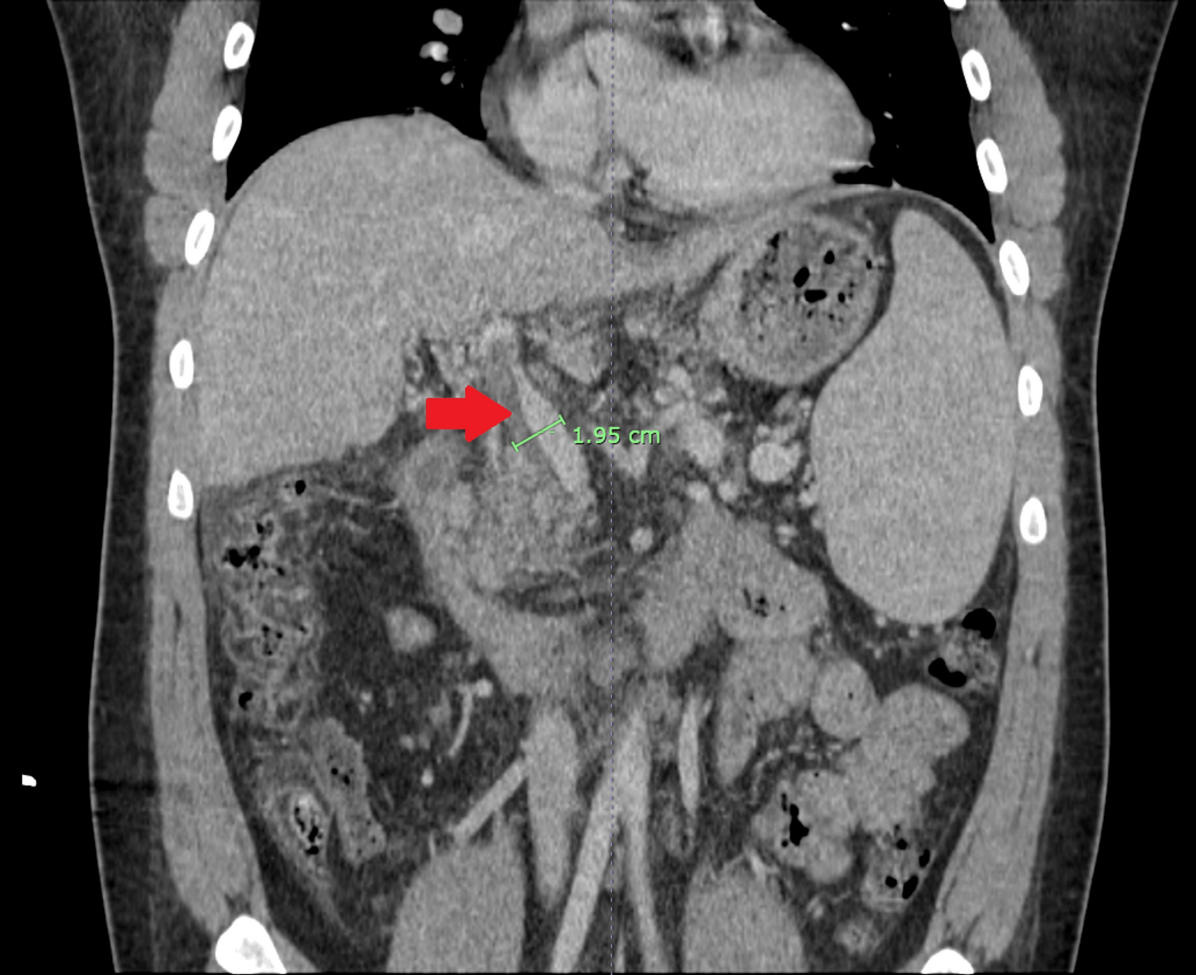
Computed tomography scan abdomen and pelvis Red arrow shows portal vein thrombus and green line shows dilated portal vein consistent with portal hypertension.

CT scan of the abdomen and pelvis with intravenous contrast showed PH and non-occlusive portal vein thrombus (Figure 1). 

He was started on pantoprazole and octreotide infusions and underwent EGD that showed severe portal hypertensive gastropathy and large esophageal varices that were banded. An extensive workup to identify any underlying hepatic or systemic disease that might be causing PH and PVT was initiated. His serum vitamin B12, folate, iron, ferritin, transferrin, iron-binding capacity, iron saturation and haptoglobulin levels were normal. Due to PVT, a hypercoaguable workup was ordered including coagulation factors II, V, VIII, IX, X levels, protein C activity, protein S activity, activated protein C resistance, Factor V Leiden mutation, and Lupus Anticoagulant, which were all normal.

In order to rule out any underlying autoimmune condition causing PH and PVT, such as systemic lupus eryhthematosis, autoimmune hepatitis, antiphospholipid syndrome, an autoimmune workup was ordered, including antinuclear antibody (ANA), beta-2-glycoprotein antibodies, anti-cardiolipin antibody, anti-liver/kidney microsomal-1 (anti-LKM-1) antibody, rheumatoid factor, anti-mitochondrial antibody (AMAs), anti-smooth muscle antibodies, which were all normal.

Other lab tests such as serum ceruloplasmin, apolipoprotein A1, alpha-2-microglobulin, alpha-fetoprotein tumor marker, JAK2 V617F mutation, JAK2 Exon12 mutation, acute hepatitis panel, human immunodeficiency virus (HIV), ebstein-barr virus (EBV), cytomegalovirus (CMV) were all normal. A fecal calprotectin was checked to rule out any possibility of underlying inflammatory bowel disease and it was elevated to 267 (Table 2).

**Table 2:** Laboratory results to identify any underlying liver or systemic disease

Lab Parameters (units)	Reference Range	Result
Vitamin B12 (pg/mL)	200 – 1100	534
Folic acid (ng/mL)	> 5.4	23.5
Iron (mg/dL)	50 – 195	52
Ferritin (ng/mL)	38 – 380	40
Iron Binding Capacity (mcg/dL)	250 – 425	270
Transferrin (mg/dL)	188 – 341	213
Haptoglobin (mg/dL)	43 – 212	164
Ammonia (UMOL/L)	0.0 – 47.0	58.2
Apolipoprotein A1 (mg/dL)	94 – 176	120
C-Reactive Protein (mg/L)	0.0 – 8.0	42.9
Alpha-2 Macroglobulin (mg/dL)	106 – 279	202
Activated Protein C Resistance (ratio)	> 2.1	4.9
Direct Russel Viper Venom Screen (ratio)	< 45	44
Factor V Leiden Mutation	Negative	Negative
Factor II (%)	70 – 150	100
Factor V (%)	65 – 150	107
Factor VIII (%)	60 – 180	172
Factor IX (%)	60 – 160	105
Factor X (%)	70 – 150	106
Lupus Anticoagulant	Negative	Negative
Protein C Activity (%)	70 – 180	85
Protein S Activity (%)	70 – 150	75
Prothrombin Gene Mutation	Negative	Negative
Ceruloplasmin (mg/dL)	14 – 40	30
Lupus Anticoagulant (ratio)	< 40	36
Antinuclear antibody	Negative	Negative
Beta-2 Glycoprotein I IgA Antibodies (unit/ml)	< 20.0	< 2.0
Beta-2 Glycoprotein I IgG Antibodies (unit/ml)	< 20.0	< 2.0
Beta-2 Glycoprotein I IgM Antibodies (unit/ml)	< 20.0	< 2.0
Cardiolipin IgG Antibody (unit/ml)	< 20.0	< 2.0
Cardiolipin IgM Antibody (unit/ml)	< 20.0	< 2.0
Liver kidney microsomal type 1 (LKM 1) IgG Antibody (units)	< 20.0	< 20.0
Rheumatoid Factor (IU/mL)	< 14	<14
Anti-mitochondrial Antibody	Not Detected	Not Detected
Anti-Smooth Muscle Antibodies (ASMA)	Not Detected	Not Detected
Alpha-Fetoprotein Tumor Marker (ng/mL)	< 6.1	1.0
Jak2 V617F mutation	Not Detected	Not Detected
JAK2 Exon12 mutation	Not Detected	Not Detected
Hepatitis A Antibody, Total	Non-Reactive	Non-Reactive
Hepatitis B Core Antibody, Total	Non-Reactive	Non-Reactive
Hepatitis C Antibody, Total	Non-Reactive	Non-Reactive
Human Immunodeficiency Virus (HIV) Ag/Ab	Non-Reactive	Non-Reactive
Epstein-barr virus (EBV) serology	Non-Reactive	Non-Reactive
Cytomegalovirus (CMV) serology	Non-Reactive	Non-Reactive
Fecal Calprotectin (mcg/g)	<50	369

Because the extensive workup performed to rule out any underlying systemic or hepatic disorder for PH came back as negative, a liver biopsy was planned. The patient underwent transjugular liver biopsy with measurement of portosystemic pressures. His hepatic venous pressure gradient was 17 mmHg, consistent with clinically significant PH. Liver pathology was negative for cirrhosis and negative for increased iron or copper deposition. The etiology of his PH and PVT remained unclear. No underlying systemic or hepatic disorder could be identified to explain his PH and PVT, hence a diagnosis of INCPH was made as a diagnosis of exclusion. He was started on carvedilol 6.25 mg twice daily for esophageal varices prophylaxis, apixaban 5 mg twice daily for PVT, pantoprazole 40 mg daily, and iron tablets. He was discharged in a stable condition with plans to repeat EGD in 4 weeks for reassessment of esophageal varices and colonoscopy to rule out any underlying inflammatory bowel disease due to elevated calprotectin levels. He was scheduled for a repeat CT scan of the abdomen in 8 weeks to reassess portal vein clot burden.

**Figure 2:**
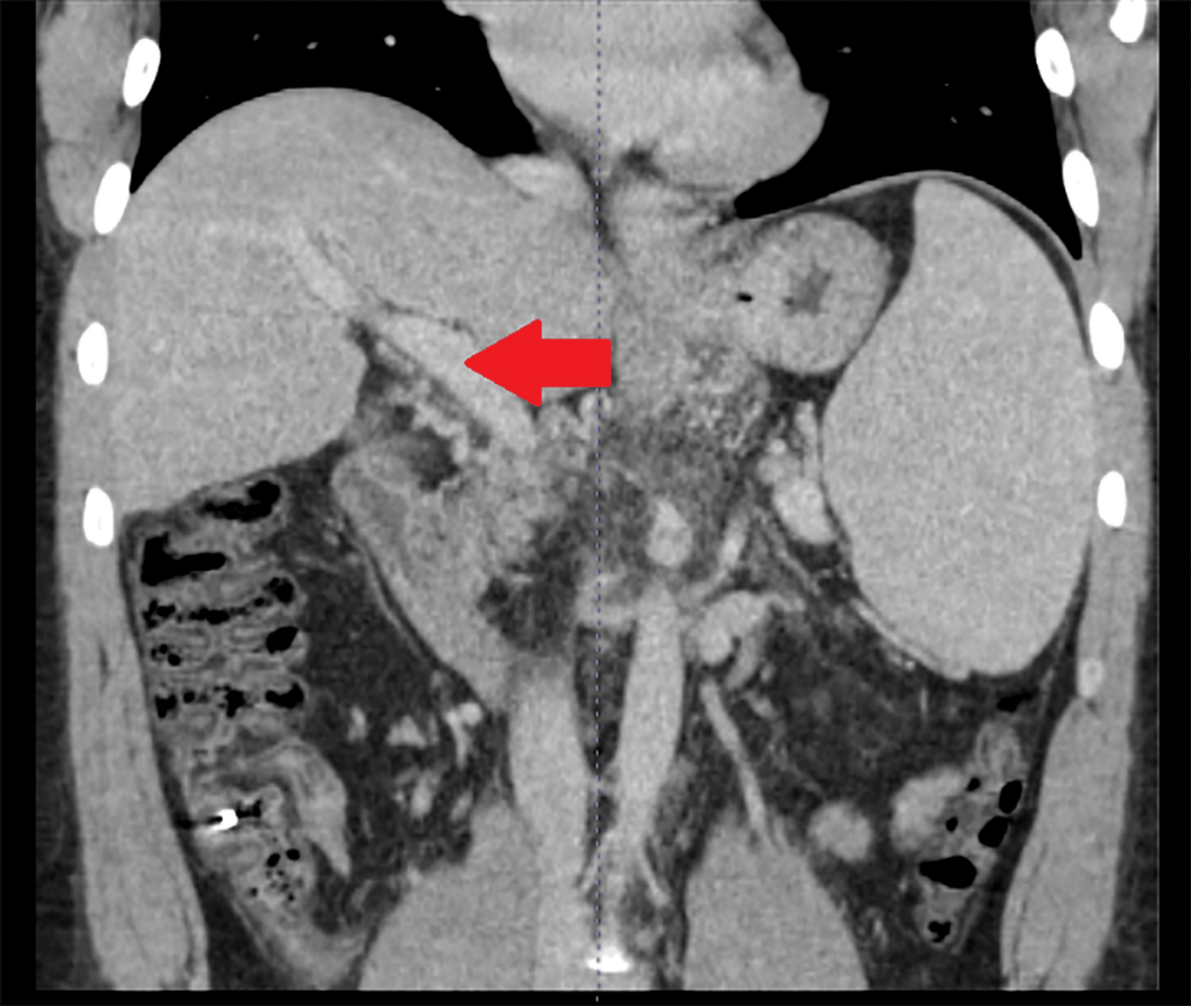
Computed tomography scan abdomen and pelvis 8 weeks after the TIPS procedure Red arrow shows improved clot burden after TIPS and anticoagulation. TIPS: Transjugular Intrahepatic Portosystemic Shunt

Repeat EGD 4 weeks later showed large (>5 mm) esophageal varices with no stigmata of bleeding, which were banded again. His colonoscopy was benign and biopsies revealed normal colonic mucosa. Due to recurrent large esophageal and gastric varices and previous life-threatening upper gastrointestinal bleeding, he was referred to an interventional radiologist for TIPS procedure for variceal embolization and/or portomesenteric thrombectomy, if indicated. He underwent successful right hepatic vein to right portal vein TIPS, and post-procedure, his portosystemic pressure gradient improved to 6 mmHg, and his varices were completely decompressed. His portal venous system did not require thrombectomy. His follow-up CT scan of the abdomen with intravenous contrast 8 weeks later showed improved overall clot burden (Figure 2). Outpatient follow-up shows he is doing well.

## Discussion

INCPH is defined as the presence of PH in a patient without any clinical evidence of underlying hepatic or systemic disease [2]. The most common clinical manifestation of INCPH is variceal bleeding and splenomegaly. Ascites and hepatic encephalopathy are less frequently seen [2 - 7]. Patients usually have near normal liver chemistry and synthetic function [2, 8, 9].

The diagnosis of INCPH is established by a liver biopsy that rules out cirrhosis and by the exclusion of any other underlying liver or systemic disease that might be causing PH, such as nonalcoholic and alcoholic steatohepatitis, Budd-Chiari syndrome, schistosomiasis, hepatic sarcoidosis, and other autoimmune disorders and vasculitides. Liver biopsy is the gold standard to rule out cirrhosis [10]. Patients also undergo hepatic venous pressure gradient (HVPG) measurement, the gold standard for diagnosing PH [3, 11]. Pressure gradient in the portal system of more than 5 mmHg without the presence of cirrhosis or primary liver or systemic disease is suggestive of INCPH.

The exact etiology of INCPH, as the name indicates, is unknown; however, it is proposed that the pathophysiology of INCPH is an increased parenchymal vascular obstruction. The outcomes of patients with INCPH are good because liver function is intact [6].

Management of INCPH is the same as that of PH in patients with cirrhosis. These patients are at risk for variceal bleeding. If a patient has variceal bleeding, he should undergo EGD with endoscopic banding of the varices. If EGD detects large non-bleeding varices, banding should still be done prophylactically. If banding fails and the patient is at risk for life-threatening bleeding, a TIPS can be considered to lower pressures in the portal system [12, 13]. Nonselective beta blockers are also recommended. Anticoagulation is recommended in cases of portal vein thrombosis. Liver transplantation is uncommon in INCPH because patients typically have fairly good liver function. Liver transplantation is only considered in cases of unmanageable complications of portal hypertension or liver failure.

Our case report is important as we present a rare case of INCPH in a young male with no significant underlying hepatic or systemic disease. INCPH is a rare disease with increasing prevalence in developed countries and needs more literature for better understanding of the disease process and management. Our case report is an important addition to the limited literature available on INCPH and will be useful for future investigators for their literature or systemic reviews on INCPH. We highlight the extensive workup required to rule out any underlying systemic and hepatic disease prior to making the correct diagnosis of INCPH, and our article will help healthcare providers manage their patients with INCPH. 

## Conclusions

INCPH is a rare disease with increasing incidence and prevalence in developed countries. The most common presentation is gastrointestinal hemorrhage, and imaging findings are concerning for PH, PVT, splenomegaly, and sometimes ascites. In such patients who have findings of PH, an extensive workup should be undertaken to rule out any systemic or hepatic causes of PH before making the diagnosis of INCPH. Patients with life-threatening variceal bleeding should undergo variceal banding, and patients whose varices are refractory to esophageal band ligation should undergo the TIPS procedure.
